# Prenatal Evaluation of MicroRNA Expressions in Pregnancies with Down Syndrome

**DOI:** 10.1155/2016/5312674

**Published:** 2016-03-24

**Authors:** Biray Erturk, Emin Karaca, Ayca Aykut, Burak Durmaz, Ahmet Guler, Baris Buke, Ahmet Ozgur Yeniel, Ahmet Mete Ergenoglu, Ferda Ozkinay, Mehmet Ozeren, Mert Kazandi, Fuat Akercan, Sermet Sagol, Cumhur Gunduz, Ozgur Cogulu

**Affiliations:** ^1^Department of Medical Genetics, Faculty of Medicine, Ege University, 35100 Izmir, Turkey; ^2^Department of Obstetrics and Gynecology, Aegean Maternity Teaching and Training Hospital, 35110 Izmir, Turkey; ^3^Department of Obstetrics and Gynecology, Faculty of Medicine, Ege University, 35100 Izmir, Turkey; ^4^Faculty of Medicine, Department of Medical Biology, Faculty of Medicine, Ege University, 35100 Izmir, Turkey

## Abstract

*Background*. Currently, the data available on the utility of miRNAs in noninvasive prenatal testing is insufficient in the literature. We evaluated the expression levels of 14 miRNAs located on chromosome 21 in maternal plasma and their utility in noninvasive prenatal testing of Down Syndrome.* Method*. A total of 56 patients underwent invasive prenatal testing; 23 cases were carrying Down Syndrome affected fetuses, and 33 control cases carrying unaffected, normal karyotype fetuses were included for comparison. Indications for invasive prenatal testing were advanced maternal age, increased risk of Down Syndrome in screening tests, and abnormal finding in the sonographic examination. In both the study and control groups, all the pregnant women were at 17th and 18th week of gestation. miRNA expression levels were measured using real-time RT-PCR.* Results*. Significantly increased maternal plasma levels of miR-3156 and miR-99a were found in the women carrying a fetus with Down Syndrome.* Conclusion*. Our results provide a basis for multicenter studies with larger sample groups and microRNA profiles, particularly with the microRNAs which were found to be variably expressed in our study. Through this clinical research, the utility of microRNAs in noninvasive prenatal testing can be better explored in future studies.

## 1. Introduction

MicroRNAs (miRNAs) are 18–22 nucleotide long, small, noncoding RNA molecules, which have critical functions throughout the epigenetic regulation of mRNAs. Recent evidence has suggested that they play important roles in a variety of physiological and pathological processes such as cell differentiation, cell proliferation, development, apoptosis, and oncogenesis [1]. Reproductive genetic testing for a prenatal diagnosis currently involves conventional diagnostic methods including chorionic villus biopsy, amniotic fluid sampling, and fetal blood sampling. Because these diagnostic methods carry a tangible risk to the fetus, researchers have been attempting to provide accurate, noninvasive, prenatal diagnostic testing of genetic diseases for years. Circulating fetal DNA is a promising molecule for the noninvasive prenatal detection of fetal chromosomal aneuploidies and has become a challenging area of modern day research [2]. With the discovery of placental miRNAs in maternal plasma, new possibilities have emerged in the area of noninvasive prenatal diagnosis [3–5]. The first study, reported by Go et al. in 2008, described a novel in vitro method by which syncytiotrophoblast-derived RNA products representative of trisomy 21 placental RNA were screened [6]. The aim of this study was to make comparisons between chromosome 21 derived miRNAs in the maternal plasma of pregnancies both with and without Down Syndrome (DS). The expression levels of 14 miRNAs in the maternal plasma of 56 pregnancies both with and without DS were evaluated.

## 2. Materials and Methods

Following approval form from Ege University Faculty of Medicine, Research Ethics Committee, written informed consent was obtained from each subject. The study was carried out in two hospitals between December 2011 and June 2014.

The study group consisted of 23 pregnant women carrying a fetus that is affected with DS as diagnosed through karyotyping and a control group consisted of 33 pregnant women carrying a healthy fetus as determined by routine follow-up throughout their noneventful pregnancies. The ages of all the pregnant women across both groups varied between 27 and 43 years old. Exclusion criteria for both groups included having an additional congenital anomaly, genetic disease, chromosomal anomaly, or any other systemic disease or infection affecting the pregnant woman or fetus. The study group and control group both consisted of pregnancies at 17th and 18th week of gestation. Karyotype analysis, including 20 well-spread metaphases, was performed. Only cases without mosaicism were included in the study.

The subjects were divided into two groups: group 1, plasma samples of pregnant women with DS fetus, and group 2: plasma samples of pregnant women without DS fetus. Results from both groups were then evaluated and compared.

For miRNA analysis, maternal plasma was obtained from all subjects in both groups. To obtain samples, 10 mL of maternal venous blood was drawn and collected in EDTA tubes. The blood samples were centrifuged at 1600 g for 10 min, 5 mL of plasma sample was transferred to blue falcon tubes and centrifuged for the second time at 2800 g for 20 min, and 5 mL from the supernatant layer was used. The supernatant layer of the plasma was then stored at −80°C until the time of study. Trisomy 21 in the pregnant women was confirmed by karyotyping in two flasks of amniotic fluid. Real-time RT-PCR was used to detect and quantify the mature miRNA expression in the samples. Frozen material was thawed and RNA was extracted from the 10 mg of homogenized sample using PureLink® RNA Mini Kit according to the protocol for TRIzol-homogenized samples (Invitrogen). The miRNeasy RNA isolation Kit (Qiagen, Hilden, Germany) was used for the isolation and enrichment of miRNAs in accordance with the manufacturer's instructions. TaqMan® microRNA assay quantification was performed using two-step RT-PCR. In reverse transcription process, cDNA was obtained from total RNA samples in TaqMan® MicroRNA Reverse Transcription Kit using specific miRNA primers. In the second step, PCR products were amplified using TaqMan® MicroRNA Assay and TaqMan® Universal PCR Master Mix by LightCycler 480 (Roche, Mannheim, Germany).

The expressions of 14 miRNAs (hsa-let-7c, hsa-mir-125b-2, hsa-mir-155, hsa-mir-3118, hsa-mir-3156, hsa-mir-3197, hsa-mir-3648, hsa-mir-3687, hsa-mir-4327, hsa-mir-4759, hsa-mir-4760-3p, hsa-mir-548x, hsa-mir-802, and hsa-mir-99a), located on chromosome 21, were evaluated. For normalization control, u6-snRNA was used.

For the statistical analysis, miRNA expressions were calculated by using RT^2^ Profiler PCR Array Software (SABiosciences http://pcrdataanalysis.sabiosciences.com/pcr/arrayanalysis.php) and a *p* value < 0.05 was considered statistically significant. The data was normalized by geometric mean to the U6 snRNA expression and a threshold cycle (Ct) cut-off was set at 35 cycles. miRNA expression interpreted from Ct of each miRNA was normalized to Ct of U6 snRNA (ΔCt = Ct miRNA − Ct U6 snRNA). ΔCt values of miR-3156 and miR-99a had normal distribution (Shapiro-Wilk test; *p* = 0.284, *p* = 0.076, resp.).

## 3. Results

A total of 56 pregnant women were included in the study. The women were all between 27 and 43 years, with a median age of 39 years in group 1 and a median age of 37 years in group 2. The mean ages of group 1 and group 2 were 37.00 ± 4.43 and 36.73 ± 3.91, respectively. In group 1, the most common indication for prenatal diagnosis was advanced maternal age (AMA) (*N*: 16; 69.6%), which was followed by high risk in maternal plasma screening (*N*: 5; 21.7%) and abnormal finding in the sonographic examination (*N*: 2; 8.7%).

In the control group, the most common indication for prenatal diagnosis was AMA (*N*: 25; 75.8%), followed by high risk in maternal plasma screening (*N*: 8; 24.2%).

**Figure 1:**
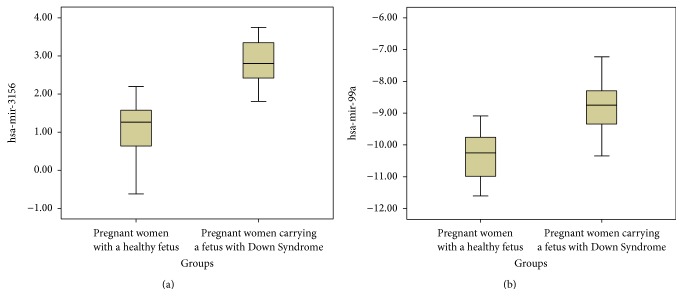
Box plots of significant miRNAs according to ΔCt values.

In brief, the plasma levels of miR-99a and miR-3156 were found to be significantly dysregulated compared to women carrying a baby without DS. Fold changes of significant miRNA expressions in relation to the study and control groups are summarized in Table 1.  Figure 1 shows the box plot of significant miRNAs.

## 4. Discussion

miRNAs have obtained great attention in the field of prenatal diagnosis following the discovery of their biological role in human development. Extracellular miRNAs that originate in trophoblasts are packaged into extracellular exosome vesicles and released into the maternal blood [7, 8]. Exosomes play an essential role in fetal development and they are found in significantly larger quantities in pregnant women when compared to nonpregnant women [9]. miRNAs can also be released from the trophoblasts as microvesicles, apoptotic bodies, and protein-bound miRNAs instead of exosomes [7]. Circulating miRNAs gain relative stability and protection from digestion by RNase, with the help of the forms previously described [2]. This durability makes them a potential biomarker. Researchers are already suggested them as a new class of circulating nucleic acids that have the potential to serve as useful clinical biomarkers [5, 6, 10].

The results from our data were significant for miR-99a and miR-3156 in which the mean values of pregnant women with an affected fetus were noticeably higher than those of women carrying an unaffected fetus. The miRNAs expressed in significant levels are summarized in Table 1. Although the sample size is not large enough to make a truly accurate evaluation, this study is important as it demonstrates the potential use of miRNAs as biomarkers for noninvasive prenatal testing (NIPT) of DS. The logical next step would be to broaden the research by using other miRNAs related to chromosome 21 with a larger study population.

NIPT by using nucleic acids in maternal blood is a much easier and less complicated procedure than constitutional noninvasive methods currently available. Pettit et al. showed a preference of NIPT by using fetal DNA in maternal blood in pregnant women whose referral reason was AMA [11]. In conjunction with this preference, increased studies have reduced turnaround times via breakthroughs such as discovery of fetal DNA in maternal blood and the completion of human genome project. Many large-scale studies incorporating the use of fetal DNA in maternal blood have been published particularly after 2010 [12–15].

Although NIPT in its current form is specific to conditions particularly with aneuploidies including chromosomes 13, 18, and 21, further studies including single gene disorders and validation studies are needed to broaden the scope of this method and integrate it into routine clinical practice. At the same time, miRNAs have also gained attention in parallel with the studies of fetal DNA. They have also been suggested as a source of biomarkers for noninvasive aneuploidy detection [16]. A number of studies evaluating the relationship between the expression levels of miRNAs with preeclampsia, fetal congenital cardiac defects, neural tube defects, fetal growth, and trisomy 21 have been reported [16–22]. Three previous studies have provided information related to the relationship between trisomy 21 and miRNAs [20–22]. Kotlabova et al. evaluated 5 miRNAs located on chromosome 21 in 12 pregnant women with high risk of bearing DS fetuses and found no significant difference when compared to a control group (women with a normal course of gestation). However, the number of pregnancies with fetuses identified with trisomy 21 was 5; therefore, it is difficult to reach an accurate conclusion in the light of this data. On the other hand, Kamhieh-Milz et al. have suggested miRNAs as promising and stable biomarkers which may allow a reliable, cost-efficient diagnostic tool for the NIPT of DS [22]. In our study, a total of 23 pregnant women with trisomy 21 fetuses were included. Among 14 miRNAs only 2, miR-99a and miR-3156, showed any significant increase in expression levels compared to a control group of pregnant women. To the best of our knowledge, there is no information to date in the literature which highlights the relationship between miR-3156 and DS prenatally. However, miR-99a has previously been found to be overexpressed in both brain and heart tissues of individuals with DS [23, 24]. Xu et al. identified this miRNA as overexpressed in trisomic lymphocytes and indicated an association between trisomy 21 and immunological defects seen in DS [25]. Higher levels of this miRNA may be associated with abnormal conditions in DS children such as immunological, cardiovascular problems and abnormal cell proliferations [23, 24, 26].

This data could also be applied to certain genetic conditions in which genetic analysis currently yields little such as mental retardation. One limitation of this study is the, respectively, low number of cases. However, our findings may add valuable insight to the field of NIPT. When the incidence of DS (1/800) in the general population and the low number of pregnancies with DS affected fetuses that reach the second trimester are taken into consideration, it is hard to find women who have accepted invasive prenatal diagnosis and fulfilled the requirements of the inclusion criteria of our study. Nevertheless, we strongly suggest designing multicenter large-scale studies with higher number of cases by including other miRNAs to understand their full potential.

This study evaluated the expression patterns of miRNAs in DS pregnancies and provided important data for planning further larger sample size studies in different clinical conditions. Our findings highlight the potential role of miRNAs as promising novel biomarkers for NIPT of genetic related diseases.

To conclude, three inferences were drawn from this study. Firstly, by further large-scale studies, maternal plasma miRNA expression can be used in DS screening. Secondly, maternal plasma miRNA may play important roles underlying the pathogenesis of DS phenotype, and, lastly, further studies evaluating fetal miRNA in the plasma of pregnant women may prove usefulness in the detection of other fetal pathologies.

## 5. Condense

Very little data is currently available on the utility of miRNAs in noninvasive prenatal testing. We evaluated herein the expression levels of 14 miRNAs located on chromosome 21 in maternal plasma samples and their utilization in noninvasive prenatal testing for Down Syndrome. The expression patterns of miRNAs in Down Syndrome pregnancies were evaluated to provide important clinical data and to ascertain their value in larger sample size studies based on different clinical conditions in the future. Our findings highlight the potential role of miRNAs as promising novel biomarkers for noninvasive prenatal testing of genetic related diseases.

## Figures and Tables

**Table 1:** Fold changes and *p* values of significantly dysregulated miRNA expressions.

miRNA	*p* value	Fold change
mir-99a	0,0410	4,20
mir-3156	0,0079	2,57
